# Crystal structure of 1,2-bis­[(1*H*-imidazol-2-yl)methylidene]hydrazine and its one-dimensional hydrogen-bonding network

**DOI:** 10.1107/S2056989016004497

**Published:** 2016-03-31

**Authors:** Chia-Hwa Lee, Gene-Hsiang Lee

**Affiliations:** a Department of Chemistry, National Taiwan University, Taipei, Taiwan; b Instrumentation Center, National Taiwan University, Taipei, Taiwan

**Keywords:** crystal structure, imidazole derivative, hydrogen bonding, supra­molecular architecture

## Abstract

In the title compound, C_8_H_8_N_6_, two imidazolyl groups are separated by a zigzag –CH=N—N=CH– linkage. An inversion center is located at the mid-point of the N—N single bond and the complete molecule is generated by symmetry. In the crystal, each mol­ecule forms four N—H⋯N hydrogen bonds with two neighbouring mol­ecules to constitute a one-dimensional ladder-like structure propagating along the *a*-axis direction.

## Abstract 2

In the title compound, two imidazolyl groups are separated by a zigzag –CH=N—N=CH– linkage. Each mol­ecule forms four N—H⋯N hydrogen bonds with two neighbouring mol­ecules to constitute a one-dimensional ladder-like structure along the *a* axis.

## Chemical context   

Supra­molecular chemistry is a fascinating topic, and mol­ecular assemblies *via* inter­molecular non-covalent binding inter­actions (*i.e.* hydrogen bonding, ionic and π–π stacking inter­actions) have attracted much attentions in the field of crystal engineering over the last decade. In particular, hydrogen bonding, which is a powerful organizing force in designing a variety of supra­molecular and solid-state architectures (Subramanian & Zaworotko, 1994 ▸), is not only used extensively to generate numerous network structures consisting of discrete organic and organometallic compounds (Desiraju, 2000 ▸), but is also responsible for inter­esting physical properties of these supra­molecular arrangements, such as electrical, optical, magnetic, *etc*. (Bacchi & Pelagatti, 2016 ▸; Lindoy & Atkinson, 2000 ▸; Létard *et al.*, 1998 ▸).
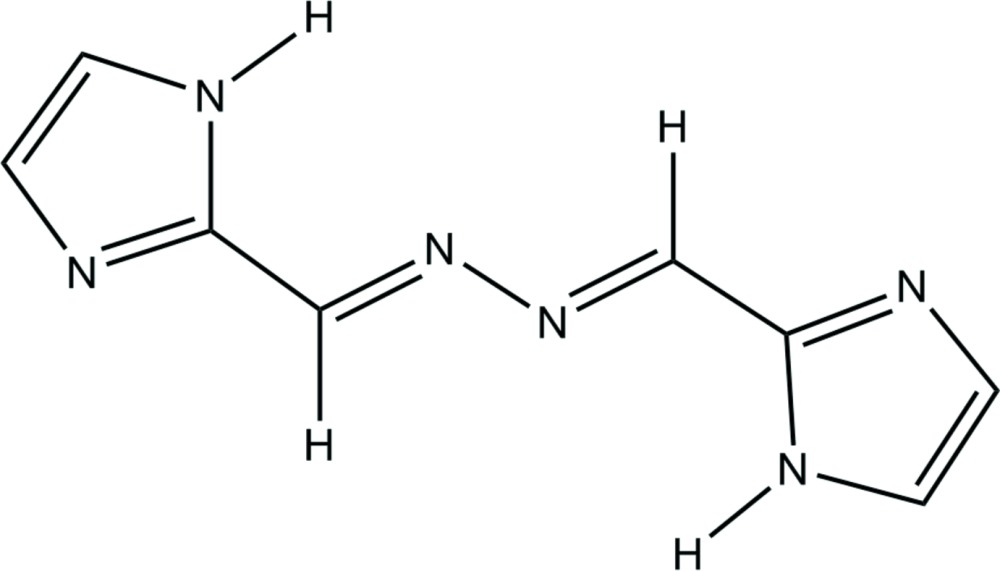


Imidazoles, containing two nitro­gen atoms, possess both hydrogen-bond donating and accepting sites and are superior building blocks for supra­molecular architectures. Many imidazole-containing polydentate ligands derived from hydrazine find a wide range of applications in coordination chemistry owing to their chelating ability (Zhou *et al.*, 2012 ▸). In this paper we report the synthesis of 1,2-bis­[(1*H*-imidazol-2-yl)methyl­ene]hydrazine (I), designed to consist of nitro­gen donors and acceptors, and the supra­molecular architecture it gives rise to *via* hydrogen bonds. The functionality of mol­ecule (I) as a bridge between metal centers for the formation of multi-dimensional structures will be discussed in subsequent publications.

## Structural commentary   

**Figure 1:**
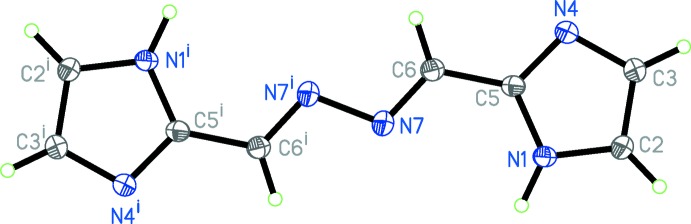
The mol­ecular structure of (I), showing the atom-labeling scheme. Displacement ellipsoids are drawn at the 50% probability level. [Symmetry code: (i) −*x*, −*y* + 1, −*z* + 2.]

The mol­ecular structure of the title compound consists of two imidazolyl groups linked by a zigzag –CH=N—N=CH– linkage (Fig. 1 ▸) and with C5⋯C5^i^ = 5.937 (3) Å [the distance between the centroids of the imidazolyl groups is 8.103 (3) Å]. The mol­ecule possesses an inversion center located in the mid-point of the N—N single bond and the complete molecule is generated by symmetry. The mol­ecule appears in a *Z*(*EE*)*Z* configuration and its geometry is similar to that of 1,2-bis­[(1*H*-imidazol-5-yl)methyl­ene]hydrazine (Pinto *et al.*, 2013 ▸) and 1,2-bis­[(thio­phene-3-yl)methyl­ene]hydrazine (Kim & Lee, 2008 ▸).

The mol­ecule (I) has a planar (r.m.s. deviation = 0.012 Å) structure which, in addition to the observed bond distances, suggests partial delocalization of the π electrons over the whole mol­ecule. The geometric parameters, *viz*., the N—N single bond [N7—N7^i^ = 1.409 (2) Å; symmetry code: (i) –*x*, −*y* + 1, −*z* + 2] , C=N double bond [C6—N7 = 1.2795 (19) Å] and C=N—N bond angle [C6=N7—N7^i^ = 111.41 (15)°], are comparable to the corresponding parameters found in 1,4-bis­(3-pyrid­yl)-2,3-di­aza-1,3-butadiene [Dong *et al.*, 2000 ▸] and 1,4-bis­(4-pyrid­yl)-2,3-di­aza-1,3-butadiene [Ciurtin *et al.*, 2001 ▸].

## Supra­molecular features   

**Figure 2:**
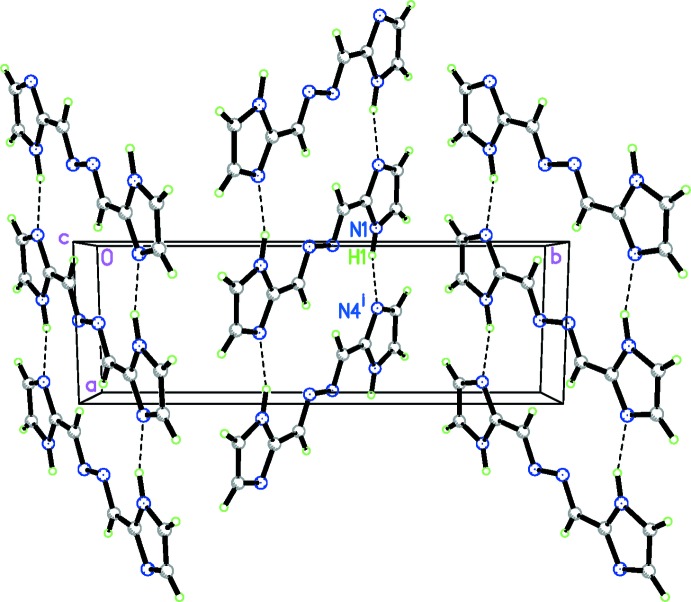
A packing diagram for (I), viewed along the *c* axis. Dashed lines represent hydrogen bonds. [Symmetry code: (i) *x* + 1, *y*, *z*.]

In the crystal structure of (I), each mol­ecule is involved in four N—H⋯N hydrogen bonds (*i.e*.: two donor and two acceptor interactions) and inter­acts with two neighboring mol­ecules, resulting in a one-dimensional ladder-like structure along the *a* axis (Fig. 2 ▸). Numerical details of the hydrogen-bonding geometry are tabulated in Table 1 ▸.

As a comparison, the related compound 1,2-bis­[(1*H*-imidazol-5-yl)methyl­ene]hydrazine (Pinto *et al.*, 2013 ▸) is a planar mol­ecule which constitutes corrugated layers parallel to the (101) plane, as a result of both hydrogen bonding and π–π stacking inter­actions with adjacent mol­ecules. In the present case of (I), instead, there are no significant π–π stacking inter­actions.

## Synthesis and crystallization   

A methanol solution (10 mL) of imidazole-2-carboxaldehyde (2.48 g, 25.8 mmol) was added to a methanol solution (10 mL) of hydrazine monohydrate (0.64 ml, 12.9 mmol). The mixture was stirred for 3 h and the precipitate was collected by filtration. Single crystals suitable for X-ray diffraction studies were obtained by diffusion of diethyl ether into a DMSO solution of the title compound (I). Yield: 2.21 g (91%).

## Refinement   

Crystal data, data collection and structure refinement details are summarized in Table 2 ▸. All the H atoms were located in difference-Fourier maps. For the H atom bounded to atom N1, the atomic coordinates and *U*_iso_ were refined, giving an N—H distance of 0.95 (2) Å. The C-bound H atoms were subsequently treated as riding atoms in geometrically idealized positions: C—H distances of 0.95 Å with *U*_iso_(H) = 1.2*U*_eq_(C).

## Supplementary Material

Crystal structure: contains datablock(s) I. DOI: 10.1107/S2056989016004497/bg2582sup1.cif

Structure factors: contains datablock(s) I. DOI: 10.1107/S2056989016004497/bg2582Isup2.hkl

Supporting information file. DOI: 10.1107/S2056989016004497/bg2582Isup3.cml

CCDC reference: 1468833

Additional supporting information:  crystallographic information; 3D view; checkCIF report

## Figures and Tables

**Table 1:** Hydrogen-bond geometry (Å, °)

*D*—H⋯*A*	*D*—H	H⋯*A*	*D*⋯*A*	*D*—H⋯*A*
N1—H1⋯N4^i^	0.95 (2)	1.95 (2)	2.8493 (17)	157.9 (19)

Symmetry code: (i) 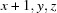.

**Table 2:** Experimental details

Crystal data	
Chemical formula	C_8_H_8_N_6_
*M*_r_	188.20
Crystal system, space group	Monoclinic, *P*2_1_/*n*
Temperature (K)	150
*a*, *b*, *c* (Å)	5.0618 (3), 14.6282 (8), 6.1294 (4)
β (°)	106.321 (2)
*V* (Å^3^)	435.56 (5)
*Z*	2
Radiation type	Mo *K*α
μ (mm^−1^)	0.10
Crystal size (mm)	0.35 × 0.10 × 0.03

Data collection	
Diffractometer	Bruker D8 VENTURE
Absorption correction	Multi-scan (*SADABS*; Bruker, 2015 ▸)
*T*_min_, *T*_max_	0.702, 0.746
No. of measured, independent and observed [*I* > 2σ(*I*)] reflections	2614, 999, 903
*R*_int_	0.014
(sin θ/λ)_max_ (Å^−1^)	0.650

Refinement	
*R*[*F* ^2^ > 2σ(*F* ^2^)], *wR*(*F* ^2^), *S*	0.042, 0.117, 1.12
No. of reflections	999
No. of parameters	68
H-atom treatment	H atoms treated by a mixture of independent and constrained refinement
Δρ_max_, Δρ_min_ (e Å^−3^)	0.31, −0.26

Computer programs: *APEX3* and *SAINT* (Bruker, 2015 ▸), *SHELXS97* and *SHELXTL* (Sheldrick, 2008 ▸) and *SHELXL2014* (Sheldrick, 2015 ▸).

## supplementary crystallographic information

### Crystal data

**Table:**

C_8_H_8_N_6_	*F*(000) = 196
*M**_r_* = 188.20	*D*_x_ = 1.435 Mg m^−^^3^
Monoclinic, *P*2_1_/*n*	Mo *K*α radiation, λ = 0.71073 Å
*a* = 5.0618 (3) Å	Cell parameters from 2074 reflections
*b* = 14.6282 (8) Å	θ = 2.8–27.5°
*c* = 6.1294 (4) Å	µ = 0.10 mm^−^^1^
β = 106.321 (2)°	*T* = 150 K
*V* = 435.56 (5) Å^3^	Needle, colourless
*Z* = 2	0.35 × 0.10 × 0.03 mm

### Data collection

**Table:**

Bruker D8 VENTURE diffractometer	903 reflections with *I* > 2σ(*I*)
φ and ω scans	*R*_int_ = 0.014
Absorption correction: multi-scan (*SADABS*; Bruker, 2015)	θ_max_ = 27.5°, θ_min_ = 2.8°
*T*_min_ = 0.702, *T*_max_ = 0.746	*h* = −6→6
2614 measured reflections	*k* = −18→19
999 independent reflections	*l* = −7→6

### Refinement

**Table:**

Refinement on *F*^2^	0 restraints
Least-squares matrix: full	Hydrogen site location: mixed
*R*[*F*^2^ > 2σ(*F*^2^)] = 0.042	H atoms treated by a mixture of independent and constrained refinement
*wR*(*F*^2^) = 0.117	*w* = 1/[σ^2^(*F*_o_^2^) + (0.0528*P*)^2^ + 0.2863*P*] where *P* = (*F*_o_^2^ + 2*F*_c_^2^)/3
*S* = 1.12	(Δ/σ)_max_ < 0.001
999 reflections	Δρ_max_ = 0.31 e Å^−^^3^
68 parameters	Δρ_min_ = −0.26 e Å^−^^3^

### Special details

**Table:**

Geometry. All e.s.d.'s (except the e.s.d. in the dihedral angle between two l.s. planes) are estimated using the full covariance matrix. The cell e.s.d.'s are taken into account individually in the estimation of e.s.d.'s in distances, angles and torsion angles; correlations between e.s.d.'s in cell parameters are only used when they are defined by crystal symmetry. An approximate (isotropic) treatment of cell e.s.d.'s is used for estimating e.s.d.'s involving l.s. planes.

### Fractional atomic coordinates and isotropic or equivalent isotropic displacement parameters (Å^2^)

**Table:**

	*x*	*y*	*z*	*U*_iso_*/*U*_eq_	
N1	−0.1374 (2)	0.62851 (8)	0.4802 (2)	0.0175 (3)	
H1	0.055 (5)	0.6193 (14)	0.515 (4)	0.037 (6)*	
C2	−0.2801 (3)	0.67725 (10)	0.2944 (2)	0.0204 (4)	
H2	−0.2082	0.7044	0.1823	0.025*	
C3	−0.5481 (3)	0.67920 (10)	0.3022 (2)	0.0197 (3)	
H3	−0.6956	0.7084	0.1937	0.024*	
N4	−0.5714 (2)	0.63279 (8)	0.4899 (2)	0.0187 (3)	
C5	−0.3193 (3)	0.60313 (10)	0.5944 (2)	0.0162 (3)	
C6	−0.2539 (3)	0.55035 (10)	0.8020 (2)	0.0180 (3)	
H6	−0.3975	0.5336	0.8659	0.022*	
N7	−0.0068 (3)	0.52574 (8)	0.9014 (2)	0.0195 (3)	

### Atomic displacement parameters (Å^2^)

**Table:**

	*U*^11^	*U*^22^	*U*^33^	*U*^12^	*U*^13^	*U*^23^
N1	0.0139 (6)	0.0223 (6)	0.0170 (6)	0.0001 (5)	0.0052 (5)	0.0008 (5)
C2	0.0187 (7)	0.0264 (8)	0.0165 (7)	0.0001 (6)	0.0055 (5)	0.0039 (5)
C3	0.0168 (7)	0.0227 (7)	0.0187 (7)	0.0013 (5)	0.0037 (5)	0.0040 (5)
N4	0.0152 (6)	0.0220 (6)	0.0188 (6)	0.0006 (5)	0.0046 (5)	0.0027 (5)
C5	0.0141 (7)	0.0180 (7)	0.0167 (7)	−0.0007 (5)	0.0049 (5)	−0.0013 (5)
C6	0.0166 (7)	0.0205 (7)	0.0173 (7)	−0.0005 (5)	0.0054 (5)	0.0004 (5)
N7	0.0198 (6)	0.0224 (6)	0.0159 (6)	0.0003 (5)	0.0043 (5)	0.0030 (5)

### Geometric parameters (Å, º)

**Table:**

N1—C5	1.3557 (18)	C3—H3	0.9500
N1—C2	1.3656 (18)	N4—C5	1.3295 (18)
N1—H1	0.95 (2)	C5—C6	1.445 (2)
C2—C3	1.371 (2)	C6—N7	1.2795 (19)
C2—H2	0.9500	C6—H6	0.9500
C3—N4	1.3689 (19)	N7—N7^i^	1.409 (2)
			
C5—N1—C2	107.26 (12)	C5—N4—C3	105.60 (12)
C5—N1—H1	130.4 (13)	N4—C5—N1	111.18 (13)
C2—N1—H1	122.3 (13)	N4—C5—C6	123.37 (13)
N1—C2—C3	106.15 (12)	N1—C5—C6	125.46 (13)
N1—C2—H2	126.9	N7—C6—C5	121.43 (13)
C3—C2—H2	126.9	N7—C6—H6	119.3
N4—C3—C2	109.81 (13)	C5—C6—H6	119.3
N4—C3—H3	125.1	C6—N7—N7^i^	111.41 (15)
C2—C3—H3	125.1		
			
C5—N1—C2—C3	−0.35 (16)	C2—N1—C5—N4	0.38 (17)
N1—C2—C3—N4	0.21 (17)	C2—N1—C5—C6	−179.95 (14)
C2—C3—N4—C5	0.02 (17)	N4—C5—C6—N7	−177.58 (13)
C3—N4—C5—N1	−0.25 (16)	N1—C5—C6—N7	2.8 (2)
C3—N4—C5—C6	−179.93 (13)	C5—C6—N7—N7^i^	−179.35 (14)

Symmetry code: (i) −*x*, −*y*+1, −*z*+2.

### Hydrogen-bond geometry (Å, º)

**Table:**

*D*—H···*A*	*D*—H	H···*A*	*D*···*A*	*D*—H···*A*
N1—H1···N4^ii^	0.95 (2)	1.95 (2)	2.8493 (17)	157.9 (19)

Symmetry code: (ii) *x*+1, *y*, *z*.
